# Neurexin1α knockout rats display oscillatory abnormalities and sensory processing deficits back-translating key endophenotypes of psychiatric disorders

**DOI:** 10.1038/s41398-022-02224-1

**Published:** 2022-10-28

**Authors:** Philipp Janz, Marie Bainier, Samuel Marashli, Philipp Schoenenberger, Miguel Valencia, Roger L. Redondo

**Affiliations:** 1 Roche Pharma Research and Early Development, Neuroscience and Rare Diseases Discovery & Translational Area, Roche Innovation Center Basel, F. Hoffmann-La Roche Ltd, Grenzacherstrasse 124, 4070 Basel, Switzerland; 2 Universidad de Navarra, CIMA, Program of Neuroscience, 31080 Pamplona, Spain; 3 IdiSNA, Navarra Institute for Health Research, 31080 Pamplona, Spain; 4 Institute of Data Science and Artificial Intelligence, Universidad de Navarra, 31080 Pamplona, Spain

**Keywords:** Molecular neuroscience, Biomarkers

## Abstract

Neurexins are presynaptic transmembrane proteins crucial for synapse development and organization. Deletion and missense mutations in all three Neurexin genes have been identified in psychiatric disorders, with mutations in the NRXN1 gene most strongly linked to schizophrenia (SZ) and autism spectrum disorder (ASD). While the consequences of NRXN1 deletion have been extensively studied on the synaptic and behavioral levels, circuit endophenotypes that translate to the human condition have not been characterized yet. Therefore, we investigated the electrophysiology of cortico-striatal-thalamic circuits in Nrxn1α^−/−^ rats and wildtype littermates focusing on a set of translational readouts, including spontaneous oscillatory activity, auditory-evoked oscillations and potentials, as well as mismatch negativity-like (MMN) responses and responses to social stimuli. On the behavioral level Nrxn1α^−/−^ rats showed locomotor hyperactivity. In vivo freely moving electrophysiology revealed pronounced increases of spontaneous oscillatory power within the gamma band in all studied brain areas and elevation of gamma coherence in cortico-striatal and thalamocortical circuits of Nrxn1α^−/−^ rats. In contrast, auditory-evoked oscillations driven by chirp-modulated tones showed reduced power in cortical areas confined to slower oscillations. Finally, Nrxn1α^−/−^ rats exhibited altered auditory evoked-potentials and profound deficits in MMN-like responses, explained by reduced prediction error. Despite deficits for auditory stimuli, responses to social stimuli appeared intact. A central hypothesis for psychiatric and neurodevelopmental disorders is that a disbalance of excitation-to-inhibition is underlying oscillatory and sensory deficits. In a first attempt to explore the impact of inhibitory circuit modulation, we assessed the effects of enhancing tonic inhibition via δ-containing GABA_A_ receptors (using Gaboxadol) on endophenotypes possibly associated with network hyperexcitability. Pharmacological experiments applying Gaboxadol showed genotype-specific differences, but failed to normalize oscillatory or sensory processing abnormalities. In conclusion, our study revealed endophenotypes in Nrxn1α^−/−^ rats that could be used as translational biomarkers for drug development in psychiatric disorders.

## Introduction

Neurexins (NRXNs) are a large family of cell adhesion proteins that play a central role in synapse maturation and organization [1] and are instrumental for Ca^2+^-dependent neurotransmission at both excitatory and inhibitory synapses [2–4]. In mammals, this family consists of three genes (Nrxn1, Nrxn2 and Nrxn3), with each gene encoding for two distinct single-pass transmembrane proteins (the larger NRXN-α and the smaller NRXN-β), both localized at the presynaptic site [5]. Through alternative splicing, thousands of structural variants can be produced, allowing NRXNs to interact with various extracellular ligands, such as neuroligins (NLGN), leucine-rich repeat transmembrane proteins, dystroglycan, neurexophilin and cerebellins [5]. This diversity is crucial for shaping synaptic function in a dynamic and circuit-specific fashion [6, 7]. For example, NRXNs cluster with heteromeric complexes of NLGN1/NLGN3 at excitatory synapses, whereas it clusters with NLGN2/NLGN3 or NLGN4 at inhibitory synapses, and dynamic insertion of splice variants is linked to activity-dependent synaptic plasticity [5, 8, 9].

The importance of NRXNs for neural circuit function is highlighted by multiple studies that have identified Nrxn deletion and missense mutations in psychiatric disorders [10–12]. Among these mutations, genetic alterations disrupting Nrxn1α are associated with SZ, ASD and intellectual disability [13–15]. In this context, NRXN1α emerged as a key element for organizing protein networks in the synaptic cleft [16], and enabling Ca2+ influx in presynaptic terminals [17]. In fact, genetically-engineered human embryonic stem cells and patient-derived pluripotent stem cells with Nrxn1 deletions show deficits in excitatory neurotransmission [18, 19]. However, a recent patch-clamp study demonstrated hyperexcitability of patient-derived stem cells with deletion specifically in Nrxn1α [20], which may manifest as epilepsy on the network level [21–23]. Rodent models with deletion of Nrxn1α display synaptic deficits as well, including decreased excitatory postsynaptic currents in CA1 pyramidal cells [4], reduced inhibition from a subpopulation of hippocampal interneurons [24], reduction of thalamic and cortical excitatory drive to striatal spiny projection neurons [25], disruption of synaptic transmission from the prefrontal cortex to the amygdala, as well as impaired feedforward inhibition within the amygdala [26]. On the behavioral level, Nrxn1 KO mice and rats elicit autistic-like traits [27–29], including learning deficits, increased grooming, deficits in sensorimotor gating and altered social behavior.

While the synaptic and behavioral alterations reported above demonstrate that Nrxn1 deletions lead to a profound disruption of diverse neural circuits, the consequences on in vivo brain physiology, which may allow to bridge animal models and clinical practice, remain unknown. Therefore, we studied the circuit physiology in freely-behaving Nrxn1α^−/−^ rats, focusing on the function of cortico-striatal-thalamic circuits, which emerged as a key network disrupted in a variety of neurodevelopmental and psychiatric disorders [30–34]. Specifically, we assessed circuit function by studying (i) spontaneous oscillatory activity and their coherence across brain areas, (ii) auditory-evoked oscillations using chirp-modulated tones, (iii) auditory-evoked potentials using simple tones, as well as MMN-like responses, and iv.) oscillatory signatures associated with social interaction. Finally, we tested whether pharmacological enhancement of tonic inhibition, using the δ subunit-containing GABA_A_ receptors agonist Gaboxadol, could normalize endophenotypes indicative of network hyperexcitability.

## Materials and methods

### Animals

Main experiments were conducted on 16 Nrxn1α^−/−^ rats and 14 wild-type littermates, and auditory brainstem response (ABR) measurements on another group of 14 Nrxn1α^−/−^ and 16 wildtype rats (strain: SD-Nrxn1<tm1sage> [27], average age: 12 weeks, average weight: 400 g) bred by Charles River, France. Sample size were based on previous experiments to provide sufficient statistical power with the expected effect sizes of our readouts-of-interest. Only male rats were used, given that behavioral abnormalities are largely confined to males in this model [27]. Rats were kept in a 12 h light/dark cycle at room temperature. Food and water were provided ad libitum. All procedures were approved by the Federal Food Safety and Veterinary Office of Switzerland and conducted in strict adherence to the Swiss federal ordinance on animal protection and welfare, as well as according to the rules of the Association for Assessment and Accreditation of Laboratory Animal Care International.

### Electrode implantation

Rats were anesthetized with 4% isoflurane for 5 min in an incubation chamber and received an injection of Buprenorphine (0.2 mg/kg s.c.) for further analgesic treatment. Throughout the surgery, Isoflurane levels were kept at 2–3% using an inhalation mask. For electrocorticography stainless steel screws were placed above the frontal and parietal cortices. Coordinates (anterior-posterior, AP; medio-lateral, ML; dorso-ventral, DV) for left frontal cortex: AP = 2.5 mm, ML = −1.2, left parietal cortex: AP = −4.0 mm, ML = −3.0 mm. Two additional screws were placed as reference and ground above the cerebellum at AP = −10, ML = ± 2. For measuring local field potentials a wire electrode (FHC, Bowdoin, Maine, USA) was lowered into the right ventromedial striatum (AP = 1.1 mm, ML = 1.5 mm, DV = −6.8 mm from cortical surface). A custom micro-drive probe (Innovative Neurophysiology, Durham, North Carolina, USA) was placed above the left mediodorsal thalamus (MDT; AP = −3.3 mm, ML = −0.7 mm, DV = −4 mm from the cortical surface) with the bundle of 16 individual microwires lowered into the MDT (target DV = −5 mm from the cortical surface). Accurate electrode placement was supported by using the AngleTwo stereotaxic system (Leica Biosystems, Wetzlar, GER). The implant was fixed to the skull with bone cement (Refobacin® Bone Cement R, Zimmer Biomet, Warsaw, Indiana, USA) and secured with ultraviolet-curing resin (Luka-fix, Cat.#: D1351305; Lukadent GmbH, Schwieberdingen, GER). For perioperative analgesia 0.1 mg/kg Buprenorphine and 1 mg/kg Meloxicam were injected s.c. directly after the end of the surgery. Postoperative analgesia (1 mg/kg Meloxicam s.c. once daily) was performed for two additional days to minimize post-surgical pain.

### Pharmacology

Animals were injected with Gaboxadol (3 mg/kg and 10 mg/kg i.p.; Cat.#: T101, Sigma-Aldrich, Darmstadt, GER) or vehicle solution (99.7% of a 0.9% saline solution + 0.3% Tween20; Cat.#: 11332465001, Sigma-Aldrich). Dosing was randomized using a latin-based square design, with each animal receiving every compound/vehicle for within-subject comparison. Dosing was performed fifteen minutes before starting the data acquisition, in line with the previously established pharmacokinetics (data not shown). No blinding was performed. The duration of the washout phase between dosings was 48 h. Blood samples were collected from the tail vein approximately two hours after dosing and exposures were confirmed by Hoffmann-La Roche bioanalytics.

### Electrophysiological recording and acoustic stimulation

Electrophysiological recordings have been performed according to Janz et al. [35].

Acoustic stimuli (calibrated to 75–80 dB SPL) were presented using a TDT RZ6-A-P1 system (Tucker-Davis Technologies, Alachua, Florida, USA), time-logged with TTL pulses in the electrophysiological data. Experimental sessions were conducted as described previously in Janz et al. [35]. Chirps consisted of pure tones (carrier frequency: 5 kHz; duration: 2 s, inter-stimulus-interval: 4 s, jitter: 10 ms) modulated in amplitude from 1 to 100 Hz over the entire duration of the tone. The amplitude modulation was performed in a continuous and linear fashion (i.e. 1 Hz at the beginning, ramping up to 100 Hz until the end of the chirp). For the auditory oddball paradigm, we used 5 and 7 kHz tones (duration: 50 ms, inter-stimulus-interval: 400 ms, jitter:10 ms). Oddballs were presented with a 10% probability to elicit mismatch responses. Tones were arranged either in an ‘ascending oddball’, ‘descending oddball’ or ‘many-standards’ sequence. For the ‘many-standards’ sequence the 5 and 7 kHz tones were randomly intermingled with 8 additional tones (2, 3, 9, 11, 13, 15, 17, and 19 kHz) each presented with 10% probability.

For ABRs, 512 click tones (50 ms inter-stimulus interval) for each volume (90–10 dB SPL in 10 dB decrements) were delivered via the RZ6-A-P1 to isoflurane-anesthetized animals (5% for induction, 2.5% for maintenance). Body temperature was maintained at approximately 37 °C using a warming pad (Kent Scientific Corporation; Torrington, Connecticut, USA). ABR signals were recorded with subdermal needle electrodes (Cat.#: NS-s83018-r9-10, Rochester, Coral Springs, Florida, US) connected to a RA4PA/RA4LI amplifier system (Tucker-Davis Technologies; settings: 12 kHz sampling rate, 5 kHz low pass, 100 Hz high pass, 50 Hz notch). Signal electrode placed on the vertex and reference/ground electrodes placed under the ipsi- and contralateral ear, respectively.

### Social response assay

The test area consisted of an open field (73.5 × 73.5 cm) divided into an animal and object zone (opposite corners) and a neutral zone in between. In the animal zone, a wildtype littermate was placed into a cylinder (22 cm diameter) built of equally-spaced plexiglas bars, allowing olfactory cues and tactile interaction between animals. In the object zone, a neutral object (kitchen paper roll) was placed into the cylinder. During the habituation phase, test animals were allowed to explore the area for 10 min without the presence of the littermate or the object. During the phase when both the animal and the object is present (AO phase, 15 min.) the wildtype littermate and the neutral object were placed into their respective areas. For the post-AO phase (10 min), both the littermate and object were again removed from the area, without cleaning to preserve olfactory cues for the post phase (cleaning was performed before starting the next session). During the experiment, the location of the animal was tracked using a Noldus system (Noldus camera and EthoVision software; Noldus Inc) and electrophysiological data was recorded with an OmniPlex system (Plexon Inc, Dallas, Texas, USA). An interaction between the test animal and its littermate or the object was considered, when the head (nose point detection method with AutoSwap correction for head/tail flipping, and manual validation) of the test animal was in the direct vicinity of the respective cylinder (5 cm min distance to cylinder wall) for at least 10 s.

### Data processing and analysis

Data analysis was performed as described in Janz et al. [35]. In brief, after downsampling, smoothing and artifact removal, data was analyzed as outlined below. The signals of the 16 channels in the MDT were averaged. From video-tracking data behavioral states (moving, quiet wakefulness and inactive) were classified and registered in the electrophysiological data. Precise synchronization of the video-tracking and electrophysiological data was ensured by sending out TTL pulses every 5 min, registered as timestamps in both data types for subsequent alignment. Morlet wavelets (centered in logarithmically spaced frequencies from 1 to 256 Hz) were used to calculate power spectral densities. Additionally, the imaginary coherence was calculated between pairs of electrodes. For chirp responses, we analyzed the evoked activity by the stimulus (normalized to the pre-stimulus baseline) within the 1–100 Hz diagonal band (logarithmically spaced, resulting in 26 data points covering 1–100 Hz). For analysis of auditory-evoked potentials, average field potentials were calculated in a −50 to 250 ms time window relative to tone onset (details in Janz et al. [35]). For ABRs, averaged responses were exported into.csv files and visualized with custom python scripts. No animals were excluded for the analysis. Blinding was not applicable, since data analysis was fully automated without involving a human scorer.

### Statistical testing

Behavioral data was statistically tested with Prism 8 software (GraphPad, San Diego, California, USA), performing two-tailed unpaired Student’s *t*-test or repeated-measures (RM) two-way ANOVA with Tukey’s or Sidak’s post-test (significance level set to *p* < 0.05). Statistical testing for electrophysiological readouts was performed with paired or unpaired cluster-based permutation tests (CBPT) using custom-made scripts in Python, as described previously [35].

## Results

### Alterations in general behavior

**Fig. 1:**
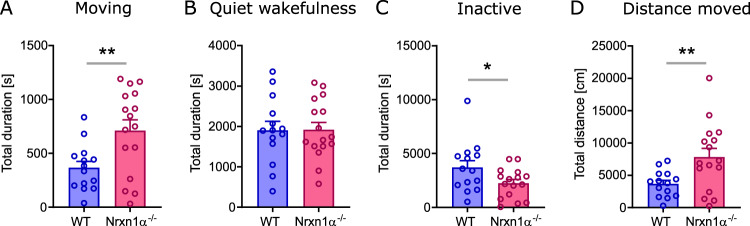
Time spent in basic behavioral states. **A** total duration of time spent moving, **B** being in a quiet wakefulness state, **C** being inactive (including sleep), and **D** total distance moved. WT (*N* = 14) displayed in blue, Nrxn1α^−/−^ (*N* = 16) shown in magenta. Each datapoint represents one animal. Data displayed as mean + SEM. Two-tailed Student’s *t*-test. *p < 0.1, **p < 0.01.

In order to assess differences in basic behavior between genotypes, we quantified the time an animal spent moving, being in a quiet wakefulness or in an inactive state during the entire experiment. Nrxn1α^−/−^ rats spent significantly more time moving (Fig. 1A; *t* = 2.92, df = 28, *p* < 0.01) and reduced time being inactive (Fig. 1C; *t* = 2.13, df = 28, *p* < 0.05), whereas there was no difference in quiet wakefulness. Furthermore, Nrxn1α^−/−^ not only spent more time moving, also the total distance moved was significantly increased (Fig. 1D; *t* = 2.78, df = 28, *p* < 0.01).

### Increased gamma power and gamma coherence

**Fig. 2:**
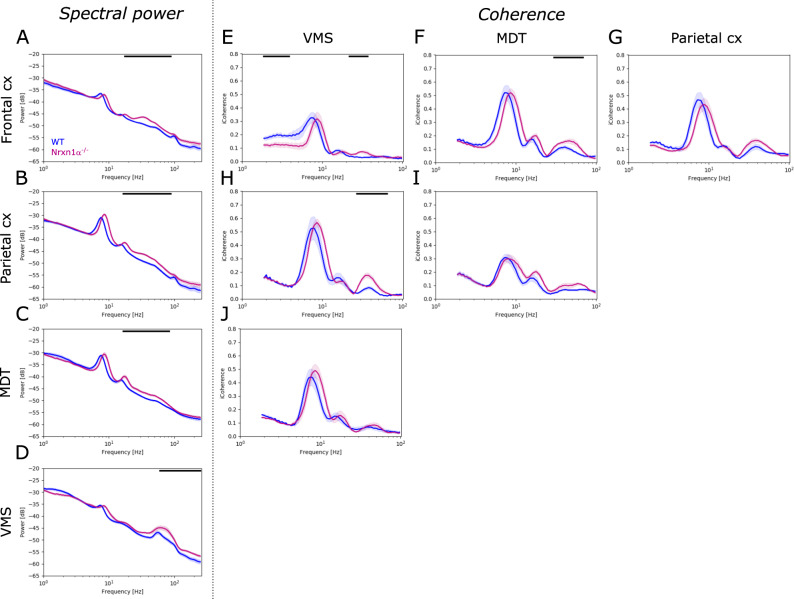
Power and coherence of spontaneous brain oscillations. **A**–**D** Spectral power of spontaneous oscillations for each brain region (frontal cortex, parietal cortex, mediodorsal thalamus, MDT and ventromedial striatum, VMS) investigated. and **E**–**J** imaginary coherence between brain regions, e.g., **E** coherence between frontal cortex and VMS, **F** between frontal cortex and MDT or **H** between parietal cortex and VMS. WT (*N* = 14) displayed in blue, Nrxn1α^−/−^ (*N* = 14) shown in magenta. Data displayed as mean + SEM and tested with unpaired CBPT. Black bars above the graphs represent clusters with statistically significant differences.

Next, we investigated spontaneous oscillatory activity in freely-moving animals, without applying sensory stimulation. Compared to their wildtype littermates, Nrxn1α^−/−^ rats showed significant elevation of oscillatory power in the gamma band across brain regions investigated. For Nrxn1α^−/−^ rats the power in broadband gamma (30–80 Hz), and in the beta frequency band (20–30 Hz), was increased in the frontal cortex (Fig. 2A; 20–100 Hz, *d* = 1.93, *p* < 0.01), in the parietal cortex (Fig. 2B; 20–90 Hz, *d* = 1.73, *p* < 0.01) and in the MDT (Fig. 2C; 20–90 Hz, *d* = 1.43, *p* < 0.01). The VMS of Nrxn1α^−/−^ rats showed increased power in high gamma and frequencies above (Fig. 2D; 80–250 Hz, *d* = 1.11, *p* < 0.01). This increase in gamma was evident across behavioral states (Supplementary Fig. 1), suggesting that it is independent from locomotor activity or brain states. Beyond the changes oscillatory power, we observed a shift of spontaneous oscillations to higher frequencies in Nrxn1α^−/−^ rats (e.g. a shift in theta peak frequency by about 0.78 Hz for the frontal and 0.87 Hz for the parietal cortex, both *p* < 0.01; data not shown).

In addition, coherence analysis revealed altered functional coupling between several brain areas of Nrxn1α^−/−^ rats compared to wildtype littermates. Particularly within the gamma band, coherence was significantly increased between the frontal cortex and VMS (Fig. 2E; 25–40 Hz, *d* = 1.95, *p* < 0.05), frontal cortex and MDT (Fig. 2F; 30–70 Hz, *d* = 1.64, *p* < 0.05) and between the parietal cortex and VMS (Fig. 2H; 30–70 Hz, *d* = 2.29, *p* < 0.001) of Nrxn1α^−/−^ rats. Beyond the increase within the gamma band, Nrxn1α^−/−^ rats showed reduced coherence in delta frequencies between the frontal cortex and VMS (Fig. 2E; 1–3 Hz, *d* = 1.72, *p* < 0.05).

In summary, Nrxn1α^−/−^ rats exhibit remarkable abnormalities in spontaneous oscillatory activity, comprising a broad increase in gamma power and increased gamma coherence in the cortico-striatal and thalamocortical circuitry.

### Reduction of auditory-evoked theta oscillations

**Fig. 3:**
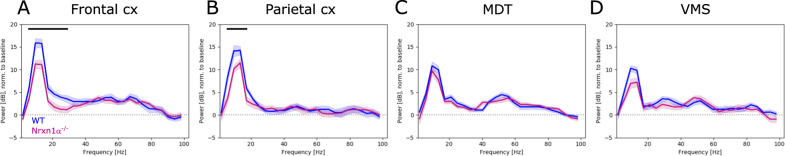
Power of auditory-evoked brain oscillations. **A** Spectral power of chirp-evoked oscillations for frontal cortex, **B** parietal cortex, **C** mediodorsal thalamus, MDT and **D** ventromedial striatum, VMS. WT (*N* = 14) displayed in blue, Nrxn1^−/−^ (*N* = 14) shown in magenta. Data displayed as mean + SEM and tested with unpaired CBPT. Black bars above the graphs represent clusters with statistically significant differences.

Having identified differences in spontaneous oscillations between genotypes, we set out to probe the capacity of brain circuits to produce sensory-evoked oscillations. Therefore, we presented chirp-modulated tones, which assess auditory-evoked oscillatory activity over a broad frequency range (1–100 Hz). Interestingly, auditory-evoked power in the gamma band (in contrast to spontaneous gamma) was not different between genotypes. Auditory-evoked power of lower frequency bands, however, was significantly reduced in the frontal cortex (Fig. 3A; 5–30 Hz, *d* = −1.09, *p* < 0.001) and the parietal cortex (Fig. 3B; 5–20 Hz, *d* = −1.05, *p* < 0.01) of Nrxn1α^−/−^ rats compared to wildtype controls. Given that evoked theta power in MDT and VMS remained similar between genotypes (Fig. 3C, D), our results highlight a cortical impairment of sensory-driven theta oscillations in Nrxn1α^−/−^ rats.

### Alterations of auditory-evoked potentials and impairment of prediction error

**Fig. 4:**
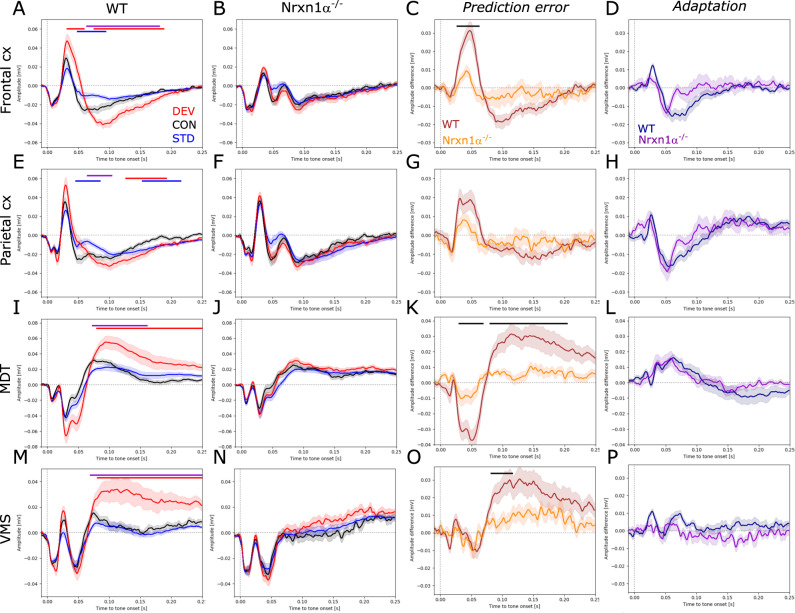
Auditory event-related potentials and mismatch responses. **A**, **E**, **I**, **M** Auditory-evoked potentials in the frontal cortex, parietal cortex, mediodorsal thalamus (MDT) and ventromedial striatum (VMS) of wildtype (*N* = 12) and **B**, **F**, **J**, **N** Nrxn1^−/−^ rats (*N* = 15). Responses to the deviant tone depicted in red, to the control tone in black and to the standard tone in blue. **C**, **G**, **K**, **O** Difference waveforms between responses to the deviant and the control tone to reveal the prediction error, and **D**, **H**, **L**, **P** difference waveforms between responses to the control and to the standard tone to assess adaptation. Data displayed as mean + SEM and tested with paired or unpaired CBPT. Colored bars (red: deviant vs. control; blue: standard vs. control; violet: deviant vs. standard; black: WT vs. Nrxn1α^−/−^) above the graphs represent clusters with statistically significant differences.

In order to investigate context-dependent sensory processing with a focus on novelty detection, we performed auditory oddball experiments. To exclude potential effects of movement, only responses that were elicited when the animal was still were analyzed. Wildtype rats showed enhanced responses to the unexpected deviant tone and reduced responses to the expected standard tone compared to the control tone, indicative of sensory adaptation and novelty detection. Significantly elevated responses to deviants were evident in the frontal cortex (Fig. 4A) between 40–60 ms (*d* = 1.34, *p* < 0.05) and 80–190 ms (*d* = 1.34, *p* < 0.001) after tone onset. Significant reduction of standard responses in the frontal cortex was confined to 50–100 ms after tone onset (*d* = −1.24, *p* < 0.05). Similar context-dependent modulation of evoked responses was also observed in the parietal cortex (Fig. 4E; increased deviant: 140–190 ms, *d* = 1.13, *p* < 0.01, reduced standard: 50–90 ms, *d* = −1.22, *p* < 0.05). Subcortical brain areas also displayed increased responses to the deviant (Fig. 4I; MDT: 80–250 ms, *d* = 1.3, *p* < 0.01; and Fig. 4M; VMS: 80–250 ms, *d* = 1.21, *p* < 0.01), but no amplitude reduction to standard tones. Remarkably, in Nrxn1α^−/−^ rats none of the investigated brain areas showed significantly enhanced responses to the deviant or reduced responses to the standard tone (Fig. 4B, F, J, N). Calculating the prediction error (deviant–control) and adaptation (control–standard) component respectively, and comparing these components between genotypes, showed that deficits in mismatch responses of Nrxn1α^−/−^ rats are largely driven by impaired prediction error generation (Fig. 4C; frontal cortex: 30–70 ms, *d* = −1.47, *p* < 0.05; Fig. 4K; MDT: 30–70 ms, *d* = −1.28, *p* < 0.05; 75–200 ms, *d* = −1.21, *p* < 0.01). Impaired adaptation had only a minor impact on mismatch deficits (Fig. 4D, H, L, P). In addition, direct comparison of responses per context between genotypes revealed the presence of an ectopic peak around 70 ms after tone onset in cortical areas of Nrxn1α^−/−^ rats (Supplementary Fig. 2).

To test whether the observed auditory processing abnormalities are a consequence of alterations on the level of the brainstem, we performed ABR measurements in a separate group of animals. We found no significant differences in ABRs between genotypes demonstrating intact peripheral and basal sensory processing (Supplementary Fig. 3).

In conclusion, our findings highlight severe deficits in context-dependent auditory processing in Nrxn1α^−/−^ rats.

### Intact responses to social stimuli

**Fig. 5:**
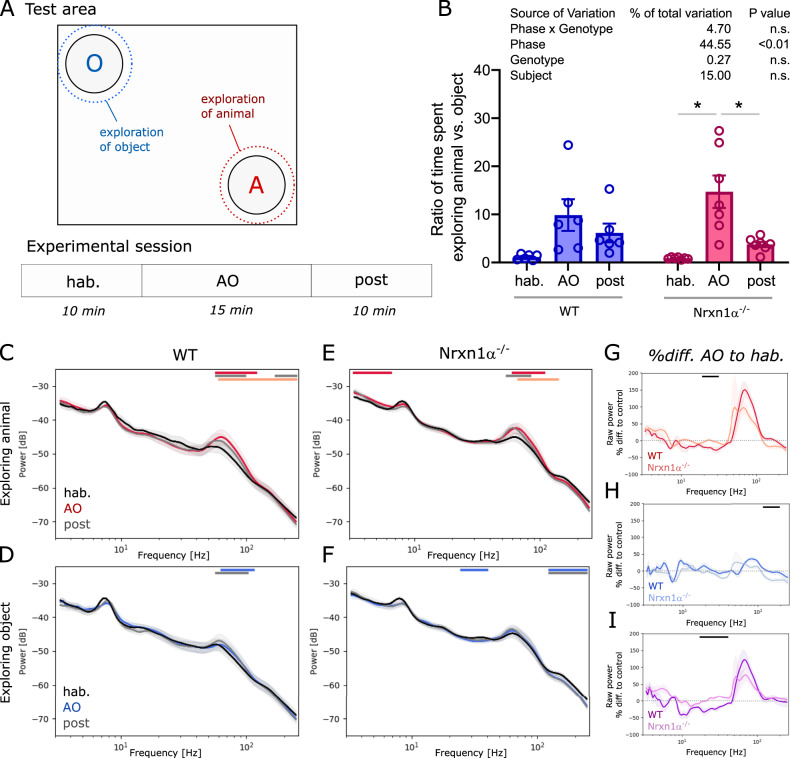
Behavior and oscillatory activity during social response assay. **A** Upper part: Schematic of test area, consisting of an open field with an object and an animal zone (rest = neutral zone) with the dotted lines indicating the area in which an encounter is scored as exploration; and lower part: Design of the experimental session (3 phases: habituation, hab; animal/object exploration, AO; post phase). **B** Box plot showing the ratio of time animals spent exploring the animal compared to exploring the object. Two-way ANOVA with Sidak’s multiple comparisons post-hoc test (*p < 0.05). Summary statistics for ANOVA results are displayed above the graphs. **C**, **D** Spectral density plots during exploration of the littermate or the object, respectively, for wildtype rats (*N* = 6) and **E**, **F** for Nrxn1α^−/−^ (*N* = 7). **G**, **H** Plots displaying the percentage difference of spectral power during animal or object exploration (normalized to the power during the habituation phase), respectively. **I** Difference plots between spectral changes during animal exploration shown in **G** and object exploration shown in **H**. Data displayed as mean + SEM and tested with paired or unpaired CBPT. Colored bars (red or blue: AO vs. hab; gray: post vs.hab; light red or light blue: AO vs. post; black: WT vs. Nrxn1α^−/−^) above the graphs represent clusters with statistically significant differences.

As Nrxn1α^−/−^ rats displayed altered responses to auditory stimuli, we hypothesized that responses to more complex and multi-modal sensory stimuli, could also be atypical. Responses to social stimuli were tested using an open field divided into a neutral zone, an animal zone and an object zone in the opposing corner (Fig. 5A). Two-way ANOVA showed that the phase of the paradigm (i.e. hab, AO and post condition) had a significant main effect on the time spent in the animal versus the object zone (Fig. 5B; 45, F_(1.201, 13.21) _= 14.68, *p* < 0.01), while no significant interaction was found between phase and genotype. Indeed, Nrxn1α^−/−^ rats spent significantly more time in the animal zone during the AO phase (both animal and object present) compared to the time spent in the same zone when no animal or object was present, i.e., during the habituation or the post-AO phase (Fig. 5B; AO vs. hab: *t* = 4.07, *p* < 0.05; AO vs. post: *t* = 3.41, *p* < 0.05). A similar trend was observed for wildtype animals. Calculating the power spectral densities for each condition revealed that the power of high gamma oscillations in the VMS of wildtype rats significantly increased, when exploring their littermates compared to the habituation phase when no littermate was present (Fig. 5C; 60–100 Hz, *d* = 0.66, *p* < 0.001), and to a lesser extent when exploring the object (Fig. 5D; 60–100 Hz, *d* = 0.28, *p* < 0.001). Interestingly, regardless of elevated power of spontaneous gamma oscillations, Nrxn1α^−/−^ rats showed a similar rise in gamma power in the VMS as observed in wildtype littermates (Fig. 5E; 3–6 Hz, *d* = 0.95, *p* < 0.05). In addition, Nrxn1α^−/−^ rats showed an increase in the power of slower oscillations during social exploring (Fig. 5E; 60–100 Hz, *d* = 0.54, *p* < 0.05). No significant elevation of gamma power was observed in Nrxn1α^−/−^ rats during exploration of the object. In contrast to wildtype animals, Nrxn1α^−/−^ rats elicited a small but significant decrease of power in low gamma and high frequency oscillations (Fig. 5F; 25–40 Hz, *d* = −0.7, *p* < 0.001; and 130–250 Hz, *d* = −0.89, *p* < 0.05). To better compare between genotypes the social interaction-driven oscillatory activity, we calculated the percentage difference of the spectra during the AO phase and the habituation phase. While there was no significant difference between genotypes for the increase in high gamma power during exploration of the littermate, we found a significant reduction of beta power in Nrxn1α^−/−^ rats (Fig. 5G; 20–30 Hz, *d* = 1.78, *p* < 0.05). This difference remained significant after normalizing the spectral densities during animal exploration with those during object exploration (Fig. 5I; 20–40 Hz, *d* = 2.75, *p* < 0.001).

Our results show that in Nrxn1α^−/−^ rats, social stimulus-driven oscillations are overall intact, but subtle attenuation of oscillatory power is evident in the beta band.

### GABA_A_δ receptor activation does not normalize aberrant oscillatory activity or auditory-evoked potentials

**Fig. 6:**
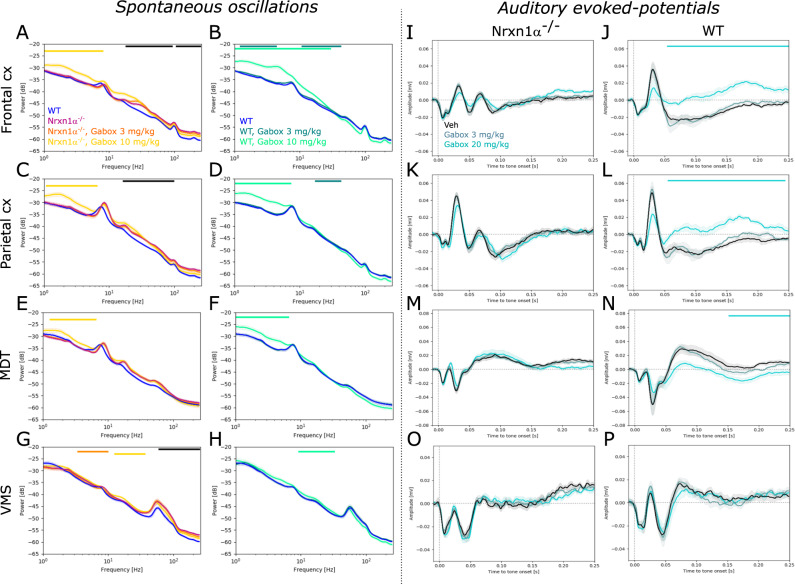
Effects of GABA_A_δ receptor-mediated tonic inhibition on oscillatory activity and auditory-evoked responses. **A**–**H** Power spectral density plots showing the effect of Gaboxadol (3 mg/kg and 10 mg/kg) on oscillatory activity in Nrxn1α^−/−^ (*N* = 10) and wildtype (*N* = 7) rats, respectively. **A**, **B** Spectrograms for frontal cortex, **C**, **D** parietal cortex, **E**, **F** MDT and **G**, **H** VMS. **I**–**P** Effect of Gaboxadol on auditory-evoked potentials in Nrxn1α^−/−^ (*N* = 10) and wildtype (*N* = 7) rats. **I**, **J** Average field potentials for frontal cortex, **K**, **L** parietal cortex, **M**, **N** MDT and **O**, **P** VMS. Data displayed as mean + SEM and tested with unpaired CBPT (for between genotype comparisons) and paired CBPT (within genotype across condition comparisons). Colored bars above the graphs represent clusters with statistically significant differences (black: Nrxn1α^−/−^ vs. WT; other colors according to the legends).

Given that some of the identified endophenotypes (increased gamma oscillations and ectopic deflections in auditory-evoked potentials) could be a consequence of increased network excitability, we tested the effect of enhancing tonic inhibition using the GABA_A_δ receptor agonist Gaboxadol in a subset of animals. In line, with our initial characterization, we found beta and gamma power significantly increased in Nrxn1α^−/−^ rats compared to their wildtype littermates (Fig. 6A; 20–90 Hz, frontal cortex: *d* = 2.02, *p* < 0.01. Fig. 6C; parietal cortex: 20–100 Hz, *d* = 2.47, *p* < 0.01. Figure 6E; trend in MDT: 30–70 Hz, *d* = 1.46; *p* = 0.066. Figure 6G; VMS: 60–250 Hz, *d* = 2.26, *p* < 0.05). Gaboxadol had no significant effect on elevated gamma power in Nrxn1α^−/−^ rats, but 10 mg/kg significantly augmented slower oscillations (<8 Hz) in both cortical regions (Fig. 6A; frontal cortex: *d* = 1.72, *p* < 0.05. Figure 6C; parietal cortex: *d* = 1.55, *p* < 0.05) and the MDT (Fig. 6E; *d* = 1.5; *p* < 0.05). Similarly, 10 mg/kg Gaboxadol significantly increased <8 Hz (and <30 Hz in the frontal cortex) oscillatory power in wildtype littermates (Fig. 6B; frontal cortex: *d* = 3.56, *p* < 0.05. Figure 6D; parietal cortex: *d* = 3.49, *p* < 0.001. Figure 6F; MDT: *d* = 1.73, *p* < 0.05).

Interestingly, auditory-evoked potentials of Nrxn1α^−/−^ rats were unaffected by Gaboxadol (except for a slight reduction of the late deflection in MDT; Fig. 6M; 200–250 ms, *d* = −0.1, *p* < 0.05), whereas significant effects were evident in wildtypes. Here, 10 mg/kg Gaboxadol treatment resulted in prolonged, positive deflections in cortical regions (Fig. 6J; frontal cortex: 60–250 ms, *d* = −3.46, *p* < 0.05; Fig. 6L; parietal cortex: *d* = −2.64, *p* < 0.05) as well as in the MDT (Fig. 6N; 160–250 ms, *d* = −1.1, *p* < 0.001). We also tested the genotype-specific effects directly, comparing Gaboxadol in Nrxn1α^−/−^ and wildtype littermate rats normalized to their respective vehicle condition (Supplementary Fig. 4). Our comparison corroborates genotype-specific effects mainly on auditory evoked potentials.

In summary, Gaboxadol did not normalize increased gamma and auditory-evoked potential abnormalities in Nrxn1α^−/−^ rats. Moreover, we found that Gaboxadol affected auditory-evoked potentials of wildtype, but not of Nrxn1α^−/−^ rats.

## Discussion

Deletion mutations of Nrxn1α have been linked to ASD, SZ and intellectual disability [10]. Considering that beyond Nrxn1α deletion mutations on a single allele, haploinsufficiency may play an important role in the pathogenesis [36], rodent models with homozygous deletion of the Nrxn1α gene elicit construct validity. Alteration in synaptic physiology and behavior associated with Nrxn1α deletion has been studied extensively, but the consequences on the level of neuronal networks remain poorly understood. Capitalizing on translational electrophysiological readouts, this study provides for the first time a characterization of circuit endophenotypes in Nrxn1α^−/−^ rats, revealing oscillatory abnormalities and deficits in auditory processing. Beyond changes in physiology, we found Nrxn1α^−/−^ rats to show hyperactivity as reported previously [27]. One of the major oscillatory abnormalities in Nrxn1α^−/−^ rats, was the augmentation of power and coherence of spontaneous gamma band oscillations. Importantly, both increased resting-state gamma power and coherence is also evident in patients with SZ [37–40] and is associated with psychotic symptoms [41–44]. Beyond its relevance in SZ and psychosis, increased gamma band power has also been reported in ASD to correlate with developmental delay [45] and increased gamma coherence has been described for Fragile X syndrome [46]. While human studies in SZ and ASD have identified elevated gamma connectivity between cortical networks using surface EEG recordings, our study’s access to intracerebral local field potentials revealed increased functional coupling within the gamma band in thalamocortical and corticostriatal circuits. In psychiatric disorders, altered functional connectivity in these circuits has been well-established by the means of fMRI. For translating our results, which are based on phase-consistency (i.e. coherence) of neuronal oscillations, it is important to consider that fMRI assesses functional connectivity based on the spatiotemporal correlation of much slower signals (e.g. blood oxygenation) that are, however, correlated with local oscillatory activity in both lower and higher frequency bands [47, 48]. In this context, fMRI studies revealed increased resting-state functional connectivity in thalamocortical networks in patients with SZ [49, 50] and ASD [51–54], as well as one study leveraging magnetoencephalography to show increased thalamocortical gamma connectivity during visual processing in SZ [55]. Recently, increased thalamocortical connectivity was discussed as central circuit dysfunction for altered sensory processing during psychosis and psychedelic states [56]. Studies focusing on corticostriatal circuits demonstrated reduced functional connectivity between the striatum and cortical regions of the salience network in both SZ and bipolar disorder [57]. In particular, reduced connectivity between ventral striatum (i.e. nucleus accumbens) and anterior cingulate cortex correlated with positive symptoms in SZ [58]. Conversely, increased connectivity between nucleus accumbens and cortical areas has been shown to be linked with hallucinations in SZ [59] and psychosis-like symptoms following Ketamine application in healthy volunteers [60]. Our study revealed decreased functional coupling between ventral striatum and frontal cortex within the theta and slow frequency band in Nrxn1α^−/−^ rats. Conversely, for the gamma band, coherence increased slightly between the striatum and cortical regions. Collectively, our results suggest altered communication in cortico-thalamic-striatal circuits of Nrxn1α^−/−^ rats, a network that is also affected in psychiatric disorders and may account for cognitive and perceptual deficits. In support of this notion, Nrxn1α^−/−^ rats display cognitive deficits in functional domains also disrupted in ASD (i.e. perception, attention, learning and executive function) [27]. Given that the cortico-striatal-thalamic network is implicated in such domains [30], our study offers a circuit-based understanding of the behavioral abnormalities described by Esclassan et al. [27].

We also tested sensory processing directly by back-translating from clinic to rodent assays that probe auditory-driven oscillations and context-dependent auditory processing (i.e. MMN), two circuit mechanisms that have been shown to be impaired in SZ [61, 62] and ASD [63–65]. For probing auditory-driven oscillations we used ‘chirps’, which entrain oscillatory activity across a broad frequency range [66, 67] and reveal oscillatory deficits in disorders such as SZ and Dravet syndrome [68, 69]. In contrast to studies performed in SZ patients, demonstrating deficits in 40 Hz auditory steady-state responses [70, 71], we found that in Nrxn1α^−/−^ rats auditory-evoked gamma oscillations appear intact, whereas the power of evoked theta oscillations was reduced. The notion that the brains of Nrxn1α^−/−^ rats have difficulties in generating theta oscillations upon sensory stimuli, is further supported indirectly by our finding that Nrxn1α^−/−^ rats display a marked impairment of mismatch responses, because mismatch responses are closely linked to evoked theta oscillations [72]. In fact, reduced mismatch responses are one of the best characterized endophenotypes in SZ and psychosis [73, 74]. Importantly, our observed auditory processing deficits are likely to be a result of higher-order circuit dysfunction, since ABRs were unaffected in Nrxn1α^−/−^ rats. While ABR abnormalities are well documented in ASD [75, 76], ABR phenotypes in SZ are inconclusive [77], indicating that disruption of auditory brainstem circuitry is not crucial for the sensory deficits observed in clinical practice.

So far we have discussed gamma oscillation abnormalities and auditory processing deficits. During social cues, striatal gamma oscillations have been described as a key signature for reward anticipation and delivery [78, 79]. Hypofunction of the ventral striatum during reward processing has been reported for SZ [80], and in particular for social stimuli in both SZ and ASD [81, 82]. Given this, we predicted that social cues could fail to induce normal gamma oscillations in Nrxn1α^−/−^ rats. Our study revealed only subtle alterations in striatal oscillatory activity during social exploration in Nrxn1α^−/−^ rats. While elevation of gamma power in the ventral striatum was not significantly affected, the concomitant reduction of power mainly within the beta band was impaired. Although there is evidence for task-related suppression of beta activity in the striatum and its relationship to dopamine release [83], its role during processing of social stimuli is unknown and beyond the scope of this manuscript.

From a mechanistic perspective, we suggest N-Methyl-d-aspartate receptor (NMDAR) hypofunction to be involved in the oscillatory abnormalities and sensory processing deficits we identified in Nrxn1α^−/−^ rats for two reasons. First, Nrxn1 has been shown to be important for postsynaptic NMDAR recruitment and function [3, 25] and second, core endophenotypes found in our study, such as increased spontaneous gamma power and deficits in MMN-like responses can be induced by pharmacological blockade of NMDAR in rat [35, 84–86], non-human primate [87–89] and human [90–92]. In this regard, our results line up with the NMDAR hypofunction hypothesis for SZ and ASD [93, 94] by linking characteristic NMDAR-dependent translational endophenotypes to disease-relevant Nrxn1α deletions with construct validity. Clearly, pathways beyond NMDAR-dependent neurotransmission can be disrupted by Nrxn1 deletions, such as GABAergic signaling [95]. Inspired by previous findings that (i) Nrxn1 deletions can lead to hyperexcitability [20–23], (ii) gamma band abnormalities in SZ likely represent deficient GABAergic interneuron function [96], and (iii) deficits in tonic inhibition have been described for other genetic-risk models for neurodevelopmental disorders [97–99], we tested the effect of enhancing tonic inhibition using Gaboxadol. We found that Gaboxadol did not alleviate gamma power or auditory-evoked potential abnormalities in Nrxn1α^−/−^ rats, suggesting that these endophenotypes are not driven by reduced tonic inhibition. Of note, our results do not argue against a disturbance of the excitation-to-inhibition balance as an underlying mechanism, considering that NMDAR hypofunction which recreates the Nrxn1α^−/−^-related endophenotypes leads to cortical disinhibition as reported previously [100]. Given that, Gaboxadol altered evoked potentials in wildtype littermates but not in Nrxn1α^−/−^ rats, it is intriguing to speculate that the lack of pharmacological modulation in Nrxn1α^−/−^ rats might be due to an already increased tonic inhibition in the model, or because of other synaptic and network deficits (such as NMDAR hypofunction) that may result in flooring effects. While this cannot be answered in the current study, our data should motivate further research in this direction.

In conclusion, our study showed for the first time that Nrxn1α^−/−^ rats display translational endophenotypes of neurodevelopmental and psychiatric disorders (most notably SZ and psychosis). The construct validity of the model and the translational nature of the readouts could support the development of novel therapies.

## Supplementary information

Supplementary figures
