# Supplementary material for: Neurexin1α knockout rats display oscillatory abnormalities and sensory processing deficits back-translating key endophenotypes of psychiatric disorders
Source: Transl Psychiatry. 2022 Oct 28;12:455. doi: 10.1038/s41398-022-02224-1 (PMC9616904; doi:10.1038/s41398-022-02224-1)
Supplement: Supplementary file 1 — Supplementary figures [file 41398_2022_2224_MOESM1_ESM.docx]

Supplementary Material

Neurexin1α knockout rats display oscillatory abnormalities and sensory processing deficits back-translating key endophenotypes of psychiatric disorders

P. Janz^1^, M. Bainier^1^, S. Marashli^1^, P. Schoenenberger^1^, M. Valencia^2,3^, R. Redondo^1^

^1^Roche Pharma Research and Early Development, Neuroscience and Rare Diseases, Roche Innovation Center Basel, F. Hoffmann-La Roche Ltd, Grenzacherstrasse 124, 4070 Basel, Switzerland. ^2^Universidad de Navarra, CIMA, Program of Neuroscience, 31080, Pamplona, Spain. ^3^IdiSNA, Navarra Institute for Health Research, 31080, Pamplona, Spain.


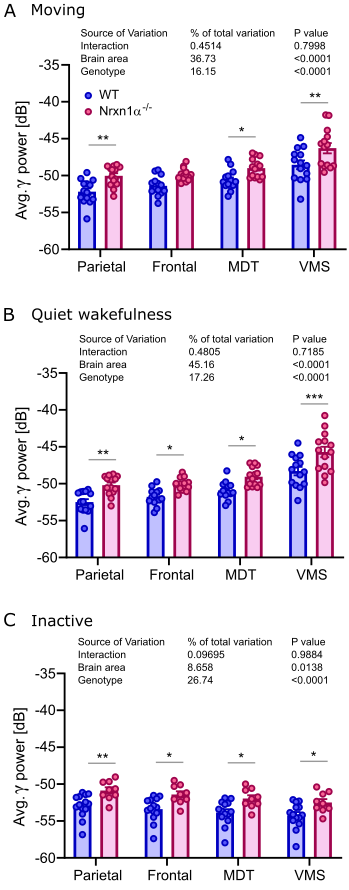


**Supplementary figure 1: Elevated gamma power is independent of the behavioral state.** A) Average gamma power for parietal cortex, frontal cortex, mediodorsal thalamus (MDT) and ventromedial striatum (VMS) during moving, B) the quiet wakefulness and C) the inactive state. WT (N=14) displayed in blue, Nrxn1a^-/-^ (N=14) shown in magenta. Each datapoint represents one rat. Two-way mixed ANOVA with Sidak’s post-test. ***p<0.001, **p<0.01, *p<0.1. Summary statistics for ANOVA results are displayed above the graphs.


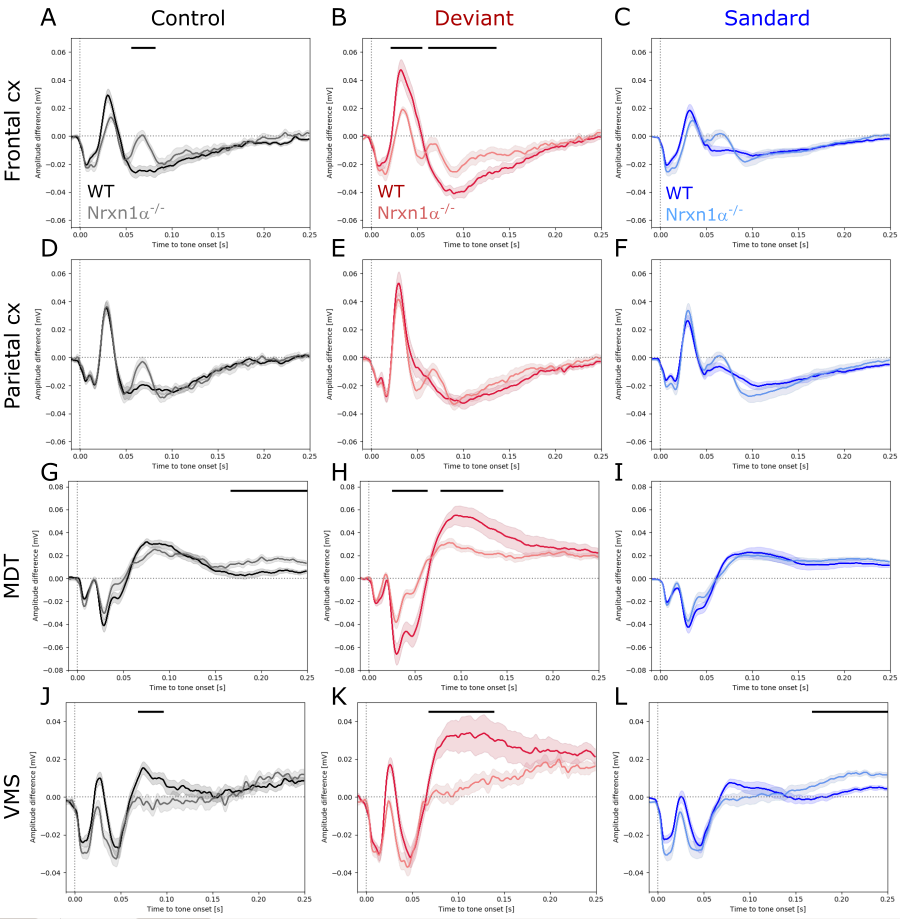


**Supplementary figure 2: Alterations of auditory event-related potentials.** A-C) Comparison of evoked potentials between genotypes (wildtype, N=12; Nrxn1a^-/-^ N=15) for each context (control responses in black; deviants in red, and standards in blue) for the frontal cortex, D-F) for the parietal cortex, G-I) for the MDT, and J-L) for the VMS. Note, the pronounced decrease in amplitude of responses upon deviant tones in Nrxn1a^-/-^ rats. Moreover, Nrxn1a^-/-^ rats display ectopic positive deflections in cortical areas peaking around 70 ms after tone onset, which is evident for each context. Data displayed as mean + SEM and tested with unpaired CBPT. Black bars above the graphs represent clusters with statistically significant differences.


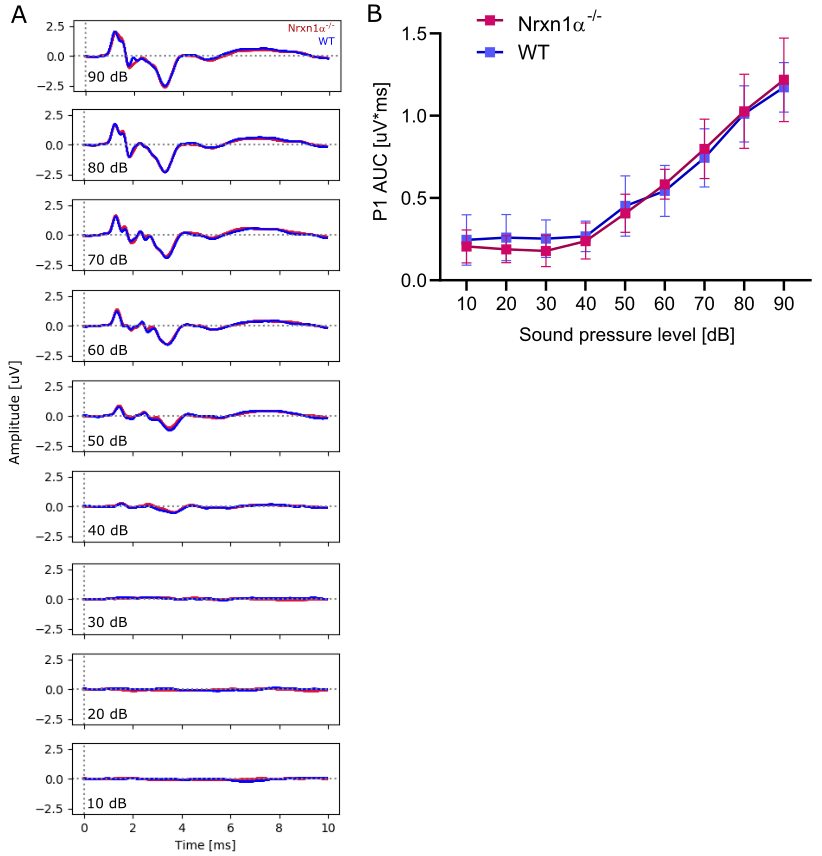


**Supplementary figure 3: Intact auditory brainstem responses.** A) Waveforms of auditory brainstem responses are similar between genotypes across stimulus intensities (10-90 dB). Responses from Nrxn1a^-/-^ (N=14) displayed in magenta, wildtypes (N=16) in blue. Tested with unpaired CBPT. B) To further study hearing thresholds, we calculated the area-under-the-curve for the first peak (P1, 1-1.7 ms time window). No statistically significant differences were observed between genotypes. Data displayed as mean + SEM. Two-way RM ANOVA with Sidak’s post-test.


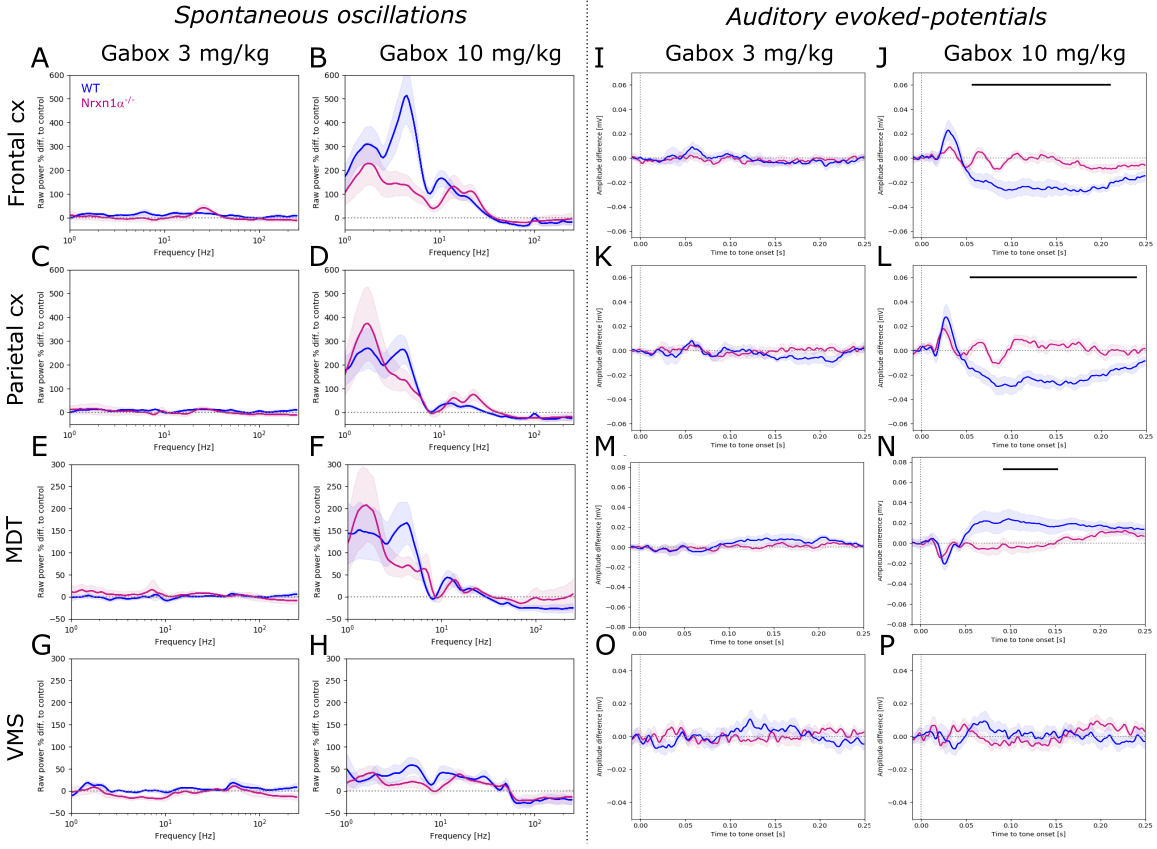


**Supplementary figure 4: Genotype-specific differences of GABA_A_δ receptor-mediated tonic inhibition on oscillatory activity and auditory-evoked responses.** A-H) Power spectral density difference plots (% difference of raw power compared to vehicle control) showing the effect of Gaboxadol (3 mg/kg and 10 mg/kg) on oscillatory activity in Nrxn1a^-/-^ (N=10) and wildtype (N=7) rats, respectively. A-B)F or frontal cortex, C-D) parietal cortex, E-F) MDT and G-H) VMS. I-P) Difference ERP plots (treatment - vehicle control) showing differential effect of Gaboxadol on auditory-evoked potentials in Nrxn1a^-/-^ (N=10) and wildtype (N=7) rats. I-J) For frontal cortex, K-L) parietal cortex, M-N) MDT and O-P) VMS. Data displayed as mean + SEM and tested with unpaired CBPT. Black bars above the graphs represent clusters with statistically significant differences.
